# Prediction of fully compensated ferrimagnetic spin-gapless semiconducting FeMnGa/Al/In half Heusler alloys

**DOI:** 10.1107/S2052252519005062

**Published:** 2019-05-09

**Authors:** Y. J. Zhang, Z. H. Liu, Z. G. Wu, X. Q. Ma

**Affiliations:** a School of Civil Engineering, Guangzhou University, Guangzhou 510006, People’s Republic of China; b Department of Physics, University of Science and Technology Beijing, Beijing 100083, People’s Republic of China

**Keywords:** FeMnGa, Heusler alloys, half-metallic fully compensated ferrimagnets, spin-gapless semiconductors, FeMn-based, full spin polarization, magnetic inversion symmetry

## Abstract

Materials with full spin polarization that exhibit zero net magnetization attract great scientific interest because of their potential applications in spintronics. Here, the structural, magnetic and electronic properties of a *C*1*_b_*-ordered FeMnGa alloy are reported using first-principles calculations. The results indicate that the corresponding band structure exhibits a considerable gap in one of the spin channels and a zero gap in the other thus allowing for high mobility of fully spin-polarized carriers. The localized magnetic moments of Fe and Mn atoms have an antiparallel arrangement leading to fully compensated ferrimagnetism, which possesses broken magnetic inversion symmetry. Such magnetic systems do not produce dipole fields and are extremely stable against external magnetic fields. Therefore, this will improve the performance of spintronic devices. Using this principle, similar band dispersion and compensated magnetic moments were predicted in a *C*1*_b_*-ordered FeMnAl_0.5_In_0.5_ Heusler alloy.

## Abstract 2

First-principles calculations suggest that *C*1*_b_*-ordered FeMn*Z* (*Z* = Al, Ga, In and Al_0.5_Ga_0.5_) alloys are half-metallic fully compensated ferrimagnets with a spin-gapless semiconducting nature. In such materials, full spin polarization, spin-gapless properties and absence of external stray fields, thus exhibiting minimal energy losses, are expected.

## 1. Introduction   

Cubic Heusler alloys are a rich family of materials that were first discovered by Heusler in 1903 (Heusler, 1903 ▸). These alloys have attracted much attention because of their wide range of extraordinary multi-functional properties including half-metallic ferromagnetism (de Groot *et al.*, 1983 ▸), thermo­electric materials (Uher *et al.*, 1999 ▸), magnetic shape memory effects (Webster *et al.*, 1984 ▸; Liu *et al.*, 2003 ▸; Zhu *et al.*, 2009 ▸) and tunable topological insulators (Lin *et al.*, 2010 ▸). Half-metallic ferromagnets (HMFs), which exhibit a complete spin polarization at the Fermi energy (*E*_F_), are a natural source of spin-polarized current and are thus considered useful for spintronic applications (de Groot *et al.*, 1983 ▸; Wolf *et al.*, 2001 ▸). A nominally infinite magneto­resistance can be achieved if the two magnetic layers of magnetic tunnel junctions (MTJs) are replaced by HMFs (de Groot *et al.*, 1983 ▸; Katsnelson *et al.*, 2008 ▸). For example, giant tunnel magneto­resistance ratios of up to 1995% were obtained for epitaxial Co_2_MnSi/MgO/Co_2_MnSi MTJs, where Co_2_MnSi is an HMF (Liu *et al.*, 2012 ▸). However, most of the ferromagnets have a major drawback: the intrinsic net dipolar moment hinders the performance of the nearby device arrays or the multilayer bits in spintronic chips (Hu, 2012 ▸). Materials with no net magnetization but high spin polarization are therefore in high demand (Hu, 2012 ▸). Obviously, this is impossible for conventional antiferromagnets (AFMs) because of the symmetry of spin rotation, which leads to no spin polarization (de Groot, 1991 ▸). Breaking the symmetry of spin rotation is then a requirement for spintronic materials in AFMs (Venkateswara *et al.*, 2018 ▸). Half-metallic antiferromagnets (HM-AFMs), which show 100% spin polarization while having zero net magnetization, are one of the most promising candidates (van Leuken & de Groot, 1995 ▸; Akai & Ogura, 2006 ▸). HM-AFMs were thought to be a special case of ferrimagnets because of the fact that they have no inversion symmetry in magnetic structure (Wurmehl *et al.*, 2006 ▸). Because of this they were later renamed half-metallic fully compensated ferrimagnets (HM-FCFs) (Wurmehl *et al.*, 2006 ▸).

In *L*2_1_ ordered *X*_2_ *YZ* Heusler alloys, when *X* and *Y* atoms carry a moment and the *X*–*Y* coupling is antiparallel, the alloys are ferrimagnets. In a carefully selected material the total magnetic moment (*M*) can be zero (Galanakis *et al.*, 2007 ▸). This is based on the famous Slater–Pauling rule, which for *X*_2_ *YZ* Heusler alloys is *M* = *N*_v_ − 24, where *N*_v_ is the number of valence electrons per formula (Galanakis *et al.*, 2002*b* ▸). The ordered *L*2_1_ structure has four formula units per cubic unit cell, with *X* in A (0, 0, 0) and C (0.5, 0.5, 0.5) sites, *Y* in B (0.25, 0.25, 0.25) sites and *Z* in D (0.75, 0.75, 0.75) sites of the Wyckoff coordinates (Cu_2_MnAl-type structure). When the valence of *Y* is larger than that of *X*, the atomic sequence is then *X*–*X*–*Y*–*Z* and the prototype is Hg_2_TiCu, which is known as an inverse Heusler alloy (Özdoğan & Galanakis, 2009 ▸). A well known example is Mn_2_CoAl (*M* = 2 *μ_B_*) (Liu *et al.*, 2008 ▸). There are three different variants, *i.e. M* = *N*_v_ − 18, *M* = *N*_v_ − 24 and *M* = *N*_v_ − 28, of the Slater–Pauling rule for the inverse Heusler alloys depending on the chemical type of the constituent transition-metal atoms (Skaftouros *et al.*, 2013 ▸ *a*). For *C*1*_b_*-ordered *XYZ* half Heusler alloys, the form of the Slater–Pauling rule is then *M* = *N*_v_ − 18. The original example of an HMF was NiMnSb (*M* = 4 *μ_B_*) (de Groot *et al.*, 1983 ▸). Quaternary Heusler alloys *XX′YZ* also obey the Slater–Pauling rule (Özdoğan *et al.*, 2013 ▸). Therefore, a generalized version of the Slater–Pauling rule can be extracted for the Heusler alloys, and half-metallic Heusler alloys with *N*_v_ = 18, 24 or 28 should be the likeliest candidates for HM-FCFs (Wurmehl *et al.*, 2006 ▸).

**Figure 1:**
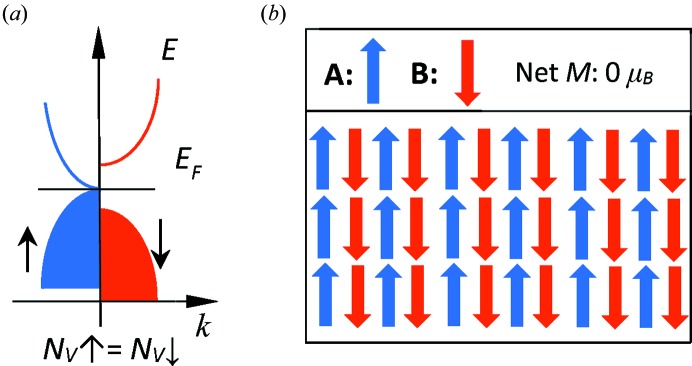
Schematic presentation of the spin-gapless semiconducting (*a*) and the compensated magnetic (*b*) properties. The compensated magnetism comes from different magnetic ions situated at sublattices A and B in an alloy.

It should be noted that in all HM-FCFs, transport is mediated by electrons having only one kind of spin, since the band structure is metallic in one spin channel. In a recently proposed new class of materials named spin-gapless semiconductors (SGSs), the realization of band gaps in both spin directions, *i.e.* an open band gap in one spin channel and a closed gap in the other, seems possible (Wang, 2008 ▸; Ouardi *et al.*, 2013 ▸). An SGS has 100% spin-polarized carriers with tunable capabilities and the speed of the fully polarized spin electrons in the SGS could be much faster than in diluted magnetic semiconductors (Wang *et al.*, 2009 ▸). As shown in Fig. 1 ▸, the SGS band structure and zero net magnetic moment are realized in the same material suggesting the discovery of a novel class of materials with both the features of HM-FCFs and SGSs. These were named fully compensated ferrimagnetic spin-gapless semiconductors (FCF-SGSs) (Jia *et al.*, 2014 ▸; Zhang *et al.*, 2015*b* ▸). Because of their unique properties, full spin polarization, higher mobility of carriers and absence of stray fields generation are expected, thus leading to minimal energy losses using FCF-SGSs as electrode materials in spintronics devices (Han & Gao, 2018 ▸; Baltz *et al.*, 2018 ▸).

To date, FCF-SGS-like properties have been observed in several Heusler ferrimagnets (Jia *et al.*, 2014 ▸), including Ti_2_- and Cr_2_-based inverse Heusler (Skaftouros *et al.*, 2013 ▸ *b*), Mn-based half Heusler (Zhang *et al.*, 2015*b* ▸), and quaternary Heusler CrVTiAl (Venkateswara *et al.*, 2018 ▸). So far few of them have been experimentally realized because generally they turned out to be unstable, or crystallized with a different structure (Feng *et al.*, 2015 ▸; Venkateswara *et al.*, 2018 ▸). The growth of the Heusler ferrimagnet Ti_2_MnAl on an Si (001) substrate has been attempted using magnetron sputtering but the samples obtained were of poor crystallinity (Feng *et al.*, 2015 ▸). The higher energy configuration CrVTiAl has a spin-gapless semiconducting nature but the corresponding configuration is not a ground state (Venkateswara *et al.*, 2018 ▸). We recently found several new FCF-SGS candidates based on the Slater–Pauling rule (Zhang *et al.*, 2015*b* ▸). But these candidates are not easy to realize in experiments. For example, *C*1*_b_*-type Mn_2_Si was predicted to be an FCF-SGS. However, Mn_2_Si alloy actually crystallized with a different structure. Therefore, the search for FCF-SGSs with a high possibility of experimental realization is still under the spotlight in spintronics.

In this study, we report that an equiatomic FeMnGa alloy with *C*1*_b_*-ordered structure can show both zero net moment and SGS properties at the same time and have a fair chance of realization. FeMnGa alloys were originally proposed as magnetic shape memory materials (Zhu *et al.*, 2009 ▸; Omori *et al.*, 2009 ▸; Liu *et al.*, 2018 ▸). Stoichiometric Fe_2_MnGa has an *L*2_1_-ordered full Heusler structure (Xin *et al.*, 2016 ▸). Although the crystal structure of stoichiometric FeMnGa has not been confirmed experimentally yet, the *C*1*_b_*-ordered structure is likely to be realized in this alloy because of two considerations. First, FeMnGa alloys crystallize to an *L*2_1_-ordered cubic phase in quite a large composition range (Omori *et al.*, 2009 ▸). Second, stoichiometric Ni_2_MnGa (Webster *et al.*, 1984 ▸) and Co_2_MnGa (Hames, 1960 ▸) are *L*2_1_-ordered full Heusler alloys, and their equiatomic forms of NiMnGa (Ding *et al.*, 2017 ▸) and CoMnGa (Wang *et al.*, 2014 ▸) were all confirmed to be *C*1*_b_*-ordered structures by experiment. In addition, competing magnetic behaviour was recently observed in Fe_41_Mn_28_Ga_31_ (Sun *et al.*, 2018 ▸). Therefore, experimental realization of *C*1*_b_*-ordered FeMnGa with zero net magnetization seems feasible. In the present work, we performed a detailed first-principles calculations study on the structural, electronic and magnetic properties of a *C*1*_b_*-ordered stoichiometric FeMnGa alloy. The results show that *C*1*_b_*-ordered FeMnGa is a potential FCF-SGS. Similar band dispersions and magnetic properties were also found in FeMnAl_0.5_In_0.5_ by first-principles calculations. Details are provided in the following sections.

## 2. Computation details and methods   

Calculations were performed using the *Vienna ab initio* simulation package (*VASP*) (Hafner, 2008 ▸). The electronic exchange and correlation used a spin-polarized generalized-gradient approximation (GGA) of density functional theory (DFT) and the electron–ion interaction was described within the framework of projector augmented-wave formalism. The wave kinetic energy cutoff was set to 500 eV and a 15 × 15 × 15 mesh for the reciprocal-space integration with a Monkhorst–Pack scheme was used for good convergence.

According to the literature, the influence of spin-orbit coupling (SOC) on the band gap of Heusler alloys is insignificant for the 3*d* series elements (Mavropoulos *et al.*, 2004 ▸). Therefore, SOC of the alloys was not employed in the calculations. For magnetism, the orbital moments originated from SOC are expected to be very small in FeMn-based alloys (Galanakis, 2005 ▸), thus the spin and orbital moments were not separated here.

The SGS properties were further confirmed by the self-consistent full-potential linearized augmented plane-wave method (Wimmer *et al.*, 1981 ▸; Weinert *et al.*, 1982 ▸). It should be pointed out that the modified Beche–Johnson (MBJ) exchange potential + local density approximation (LDA) correlation, which allows the calculation of band gaps with an accuracy similar to GW calculations, was also employed. The electronic and magnetic properties of disordered systems were calculated using the Korringa–Kohn–Rostoker (KKR) Green’s function method combined with the coherent potential approximation (CPA) (Katayama *et al.*, 1979 ▸; Blügel *et al.*, 1987 ▸; Kaprzyk & Bansil, 1990 ▸). This is a powerful method for disordered systems.

## 3. Results and discussion   

### 3.1. Crystal structure and compensated ferrimagnetism   

**Figure 2:**
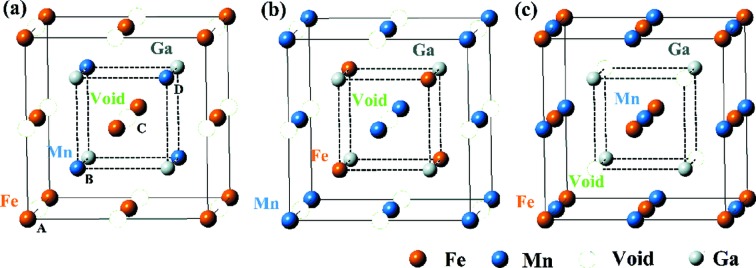
Crystal structures in conventional unit cells for three different types of Wyckoff coordinate, *i.e.* (*a*) Type I, (*b*) Type II and (*c*) Type III, for atoms in *C*1*_b_*-ordered FeMnGa alloy with 1:1:1 composition.

Half Heusler alloys with a general formula *XYZ*, where *X* and *Y* are transition metals, and *Z* is a main group element, crystallize in a non-centrosymmetric *C*1*_b_* structure. The most well known one is NiMnSb, which was found to be an HMF by de Groot *et al.* (1983 ▸). It is a ternary ordered variant of the CaF_2_ structure and can be derived from the tetrahedral ZnS-type structure by filling the octahedral lattice sites. The *C*1*_b_*-type FeMnGa alloy considered here consists of three interpenetrating f.c.c. sublattices, each of which are occupied by Fe, Mn and Ga atoms. Fig. 2 ▸ and Table 1 ▸ show the three possible inequivalent atomic arrangements and the corresponding occupied Wyckoff positions by fixing Ga at the D site.

We first performed structural optimization calculations to determine the lattice parameters and the ground state of *C*1*_b_*-ordered FeMnGa. Three possible arrangements of atoms are summarized in Table 1 ▸ and three kinds of magnetic orders, *i.e.* paramagnetic, ferromagnetic and antiferromagnetic states, are considered in the calculations. The paramagnetic (or nonmagnetic) state means that the constituent atoms of FeMnGa have no spin polarization. The ferromagnetic (or antiferromagnetic) state means the parallel (or antiparallel) alignment of the magnetic moments of the Fe and Mn atoms. Although the more precise term to describe them was thought to be compensated ferrimagnets as used in the literature (Galanakis *et al.*, 2014 ▸), the term we use here is still AFMs, which is a more general term. The moments of the *Z* atoms are usually ignored since most of them are close to zero.

**Figure 3:**
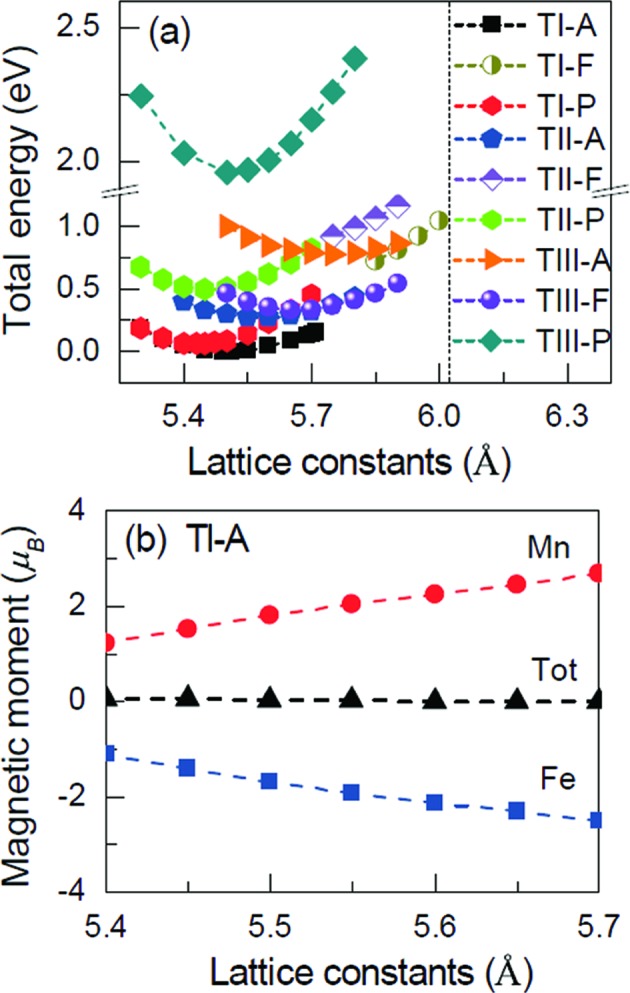
Structural optimization and fully compensated ferrimagnetism for FeMnGa. The optimized lattice constant (*a*) and the magnetic moment (*b*) are shown as functions of the lattice parameter. T*X*-P, T*X*-A and T*X*-F represent the Type-*X* paramagnetic state, the Type-*X* antiferromagnetic state, and the Type-*X* ferromagnetic state, respectively, where *X* is I, II or III.

Fig. 3 ▸(*a*) shows the calculated total energy as functions of lattice constant for FeMnGa. It can be seen that a ferromagnetic state of Type I or II configuration appears only when the lattice constants of the crystal go beyond 5.8–6.0 Å, because the ferromagneric state of FeMn cannot be stabilized in a self-consistent process below a critical distance between Fe and Mn atoms. This can be explained by the Bethe–Slater curve which shows that MnMn or MnFe atoms tend to couple antiferromagnetically when they are first nearest neighbours or the distance between them is less than a certain value (Khovaylo *et al.*, 2009 ▸). Similar situations also occur in many other Heusler alloys, such as NiMnGa, Fe_2_MnGa, Mn_2_FeGa, MnCr*Z* (*Z* = Al, Si and Sb) and FeCr*Z* (*Z* = Ga, Ge and As) (Fujii *et al.*, 2010 ▸; Zhang *et al.*, 2018 ▸). For Type III configuration [Fig. 2 ▸(*c*)], Fe and Mn atoms have a larger distance which results in a possible ferromagnetic coupling. Among all these calculated states, the Type I antiferromagnetic state is the most stable one since it has the lowest energy. This structure type agrees with the already observed half Heusler alloys (de Groot *et al.*, 1983 ▸; Galanakis *et al.*, 2002*a* ▸). The optimized equilibrium lattice constant is *a* = 5.50 Å.

Fig. 3 ▸(*b*) shows the atomic resolved and total magnetic moments of the unit cell as a function of the lattice constants. For the equilibrium lattice constant, the calculation reveals nearly full compensation of the magnetic moments. A change in the lattice parameter by up to −1.8 to 3.6% (5.4–5.7 Å) with respect to the equilibrium one only alters the local magnetic moments of the Mn and Fe atoms. An overall increase in the lattice parameter slightly increases the magnetic moment of the Mn atoms and decreases the magnetic moment of the Fe atoms. The total magnetic moment of zero, as a sum of those of each atom, remains unaffected. This result indicates the fully compensated ferrimagnetism and the half-metallic character of FeMnGa. The magnetic moment of the half-metallic *C*1*_b_* Heusler alloys follows the simple rule: *M* = *N*_v_ − 18. The robustness of the fully compensated ferrimagnetism originates from the large half-metallic gap of the minority channel that is induced by the hybridization of transition-metal atoms, as observed in many other Heusler type compounds (Galanakis *et al.*, 2002*b* ▸). The hybridization and details of the electronic structure of FeMnGa were analysed and are discussed below.

### 3.2. Electronic structure of FeMnGa   

**Figure 4:**
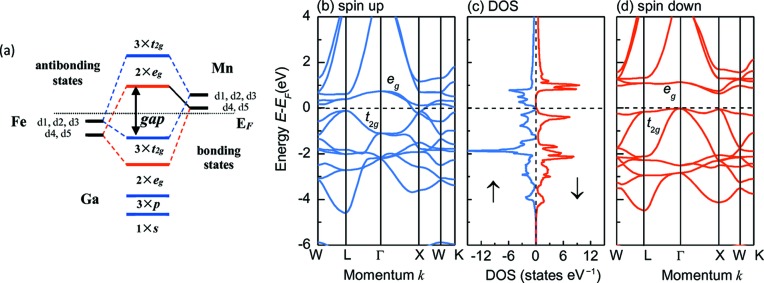
Spin-gapless semiconducting properties of *C*1*_b_*-ordered FeMnGa. (*a*) Schematic illustration of hybridization sitting at Fe and Mn sites; (*b*), (*d*) band structure; and (*c*) DOS at the equilibrium lattice constant. The band structure and DOS exhibit spin-gapless semiconducting properties and full spin polarization near the Fermi energy *E*_F_.

Detailed analyses of atomic hybridization and the origin of the half-metallic band gap in studies of Heusler-based HMFs have been performed by Galanakis *et al.* They found that, for most of the Heusler alloys, the band gap mainly arises from the hybridization of *d* states of the transition-metal atoms (Galanakis *et al.*, 2002*a* ▸,*b* ▸). The possible hybridization orbitals for *C*1*_b_* FeMnGa from low energy to high energy according to the literature are 1 × *s*, 3 × *p*, 2 × *e_g_*, 3 × *t*_2*g*__,_, 2 × *e_g_* and 3 × *t*_2*g*_ as shown in Fig. 4 ▸(*a*) (Galanakis *et al.*, 2002*a* ▸).

Figs. 4 ▸(*b*)–4(*d*) display the spin-resolved band structure and density of states (DOS) of FeMnGa at the equilibrium lattice constant. The relative band structure shows that the spin-down state has a real gap between the [*t*_2*g*_]Γ point and the [*e_g_*]Γ point with the valence band maximum (VBM) at the Γ point and the conduction band minimum (CBM) at the *X* point. Hence the gap of the spin-down state is an indirect one, which agrees with many of the *C*1*_b_*-type Heusler compounds (Zhang *et al.*, 2015*a* ▸,*b* ▸). There is also a considerable gap between the [*t*_2*g*_]Γ point and the [*e_g_*]Γ point in the spin-up channel, but the VBM and CBM are not located at the Γ point. Instead, they are located at the *L* and *X* points, respectively. In addition, the valence band and conduction band overlap at the *X* point resulting in a pseudo-gap at the Fermi level. The obtained spin-down DOS clearly shows a gap at the Fermi energy (*E*_F_), while the spin-up DOS exhibits a valley shape with the minimum value being close to zero and located at *E_F_*. This shape follows the idealized DOS for SGS (Wang, 2008 ▸). Similar electronic structure was observed in many other Heusler alloys, such as Mn_2_CoAl (Ouardi *et al.*, 2013 ▸). However, most of them have a considerable magnetic moment. FeMnGa is a very special case in which the total spin moment is exactly 0 *μ_B_*. Compared with the widely studied SGS material Mn_2_CoAl, whose magnetization is 2 *μ_B_*, FeMnGa will offer a superior advantage owing to its ideally compensated magnetic moment.

**Figure 5:**
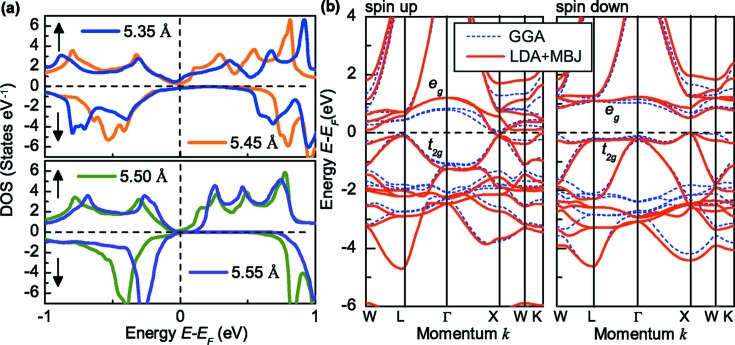
Electronic structure under uniform strain simulated by changing the lattice constants. (*a*) The total DOS for the FeMnGa alloy as a function of the lattice constant. As the lattice constant was expanded less than 1% (5.55 Å), a real closed gap was obtained. (*b*) The corresponding band structure of FeMnGa for *a* = 5.55 Å based on both the GGA and the LDA + MBJ.

Note that the closed gap of the spin-down channel is not a real zero width gap. The Fermi level cuts a little of the valence band. Uniform strain, simulated by changing the lattice constants, has often been used to open a real band gap in many Heusler alloys (Wang *et al.*, 2017 ▸). Fig. 5 ▸(*a*) shows the DOS of FeMnGa for different lattice constants. Either expansion or contraction of the lattice results in a practically rigid shift of the bands with small rearrangements of the shape of the peaks to account for the charge neutrality (Galanakis *et al.*, 2002*a* ▸). It is clearly seen that expansion by less than 1% (5.55 Å) opens a real narrow gap in the spin-up channel. Fig. 5 ▸(*b*) shows the corresponding band structure. It can be seen that both the VBM ([*t*_2*g*_]*L* point) and the CBM ([*e_g_*]*X* point) in the spin-up channel touch the Fermi level and form a zero width gap, while the VBM and CBM of the spin-down channel form an open gap with a width of ∼0.5 eV. This clearly follows the idealized band structure for SGSs.

It should be pointed out that the band structures based on the LDA + MBJ [see Fig. 5 ▸(*b*) red solid line] are similar to those based on the GGA [see Fig. 5 ▸(*b*) blue dashed line] and show SGS properties. As summarized in Table 2 ▸, contraction results in a small net magnetic moment because of the destroyed spin-down gap, while expansion retains the zero net magnetic moment. This indicates that FeMnGa has the potential for the realization of FCF-SGS.

From the discussion above, we can see that the fully compensated ferrimagnets with spin-gapless semiconducting nature can arise with net spin *N*_s_ = 0 as the number of up and down electrons are equal. Such materials have the advantage of being able to generate fully spin-polarized current while exhibiting zero net magnetization. This is very different from a conventional AFM, in which the vanishing of the net magnetic moment originates from a symmetry relation between sites of opposite spin. It causes the electronic structure of the two spin directions to be identical, which results in no polarization of the conduction electrons (de Groot, 1991 ▸). In the magnetic structure of FeMnGa, as shown in Table 2 ▸, the cancellation of the magnetic moment is not caused by a symmetry relation between sites with up and down spins, instead the moments of Fe are cancelled by the moments of Mn at different crystallographic sites. It can be seen that the magnetic moments of Fe atoms are not equal to those of Mn atoms. Ga atoms also carry a small spin magnetic moment indicating the breaking of magnetic inversion symmetry. This is one of the features of fully compensated ferrimagnetism (Venkateswara *et al.*, 2018 ▸).

**Figure 6:**
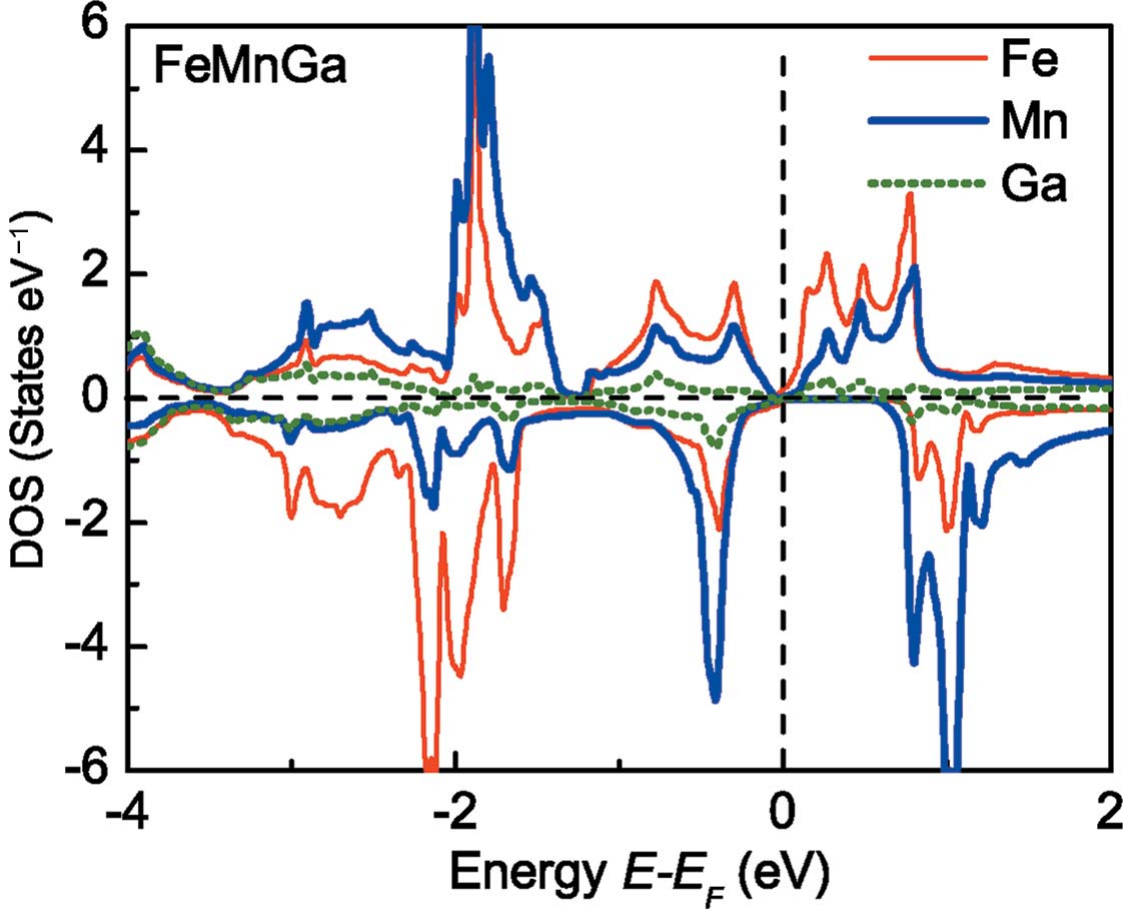
Calculated atom- and spin-resolved DOS for FeMnGa alloys. The vertical line indicates the Fermi level, and the lattice constant is taken as 5.55 Å.

Another important feature of FeMnGa that differs from conventional AFMs is that the electronic structures for the two spin directions are completely asymmetrical with respect to the spin direction, as shown in the DOS of Fig. 4 ▸(*c*). This is because the distributions of electronic states of Fe and Mn atoms in FeMnGa as a function of energy are totally different from each other. Fig. 6 ▸ shows the atom-resolved DOS, which demonstrates that the local DOS of Fe and Mn have a totally different distribution and do not completely compensate each other resulting in a spin polarization.

From the atom-resolved DOS of FeMnGa as shown in Fig. 6 ▸, it can be seen that the contribution of Ga is not dominant in forming the total DOS of the alloy. However, Ga plays a very important role in forming the SGS property in FeMnGa, as observed in many other alloys (Galanakis *et al.*, 2002*a* ▸). This is because the Ga atom has three valence electrons, and in FeMnGa each Ga atom introduces a deep lying *s* band, and three *p* bands below the centre of the *d* bands. These bands accommodate a total of eight electrons per unit cell, so that formally Ga acts as a five-charged Ga^5−^ ion. Analogously, an Sb atom behaves in NiMnSb as an Sb^3−^ ion. This does not mean that locally such a large charge transfer exists. In fact, the *s* and *p* states strongly hybridize with the transition-metal *d* states, and the charge in these bands is delocalized and locally Ga loses only about one electron, if one counts the charge in the Wigner–Seitz cells. What does count is that the *s* and *p* bands accommodate eight electrons per unit cell, thus effectively reducing the *d* charge of the transition-metal atoms. This will have a crucial effect on the distribution of the density state of each magnetic atom, hence affecting the total DOS of the alloy.

**Figure 7:**
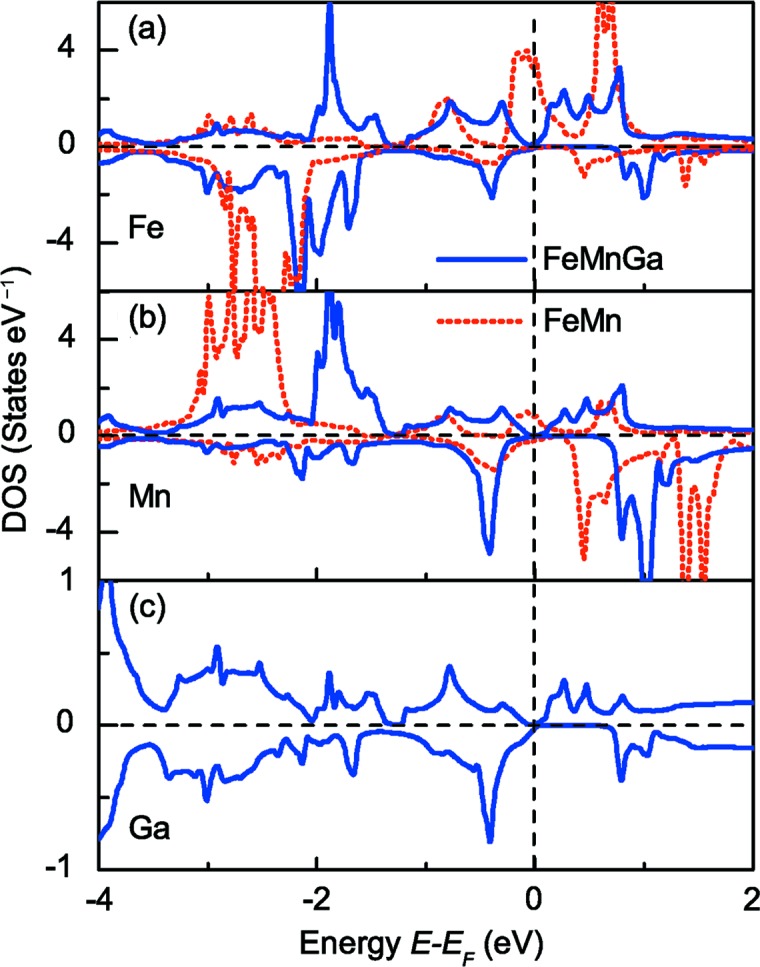
Influence of *p*-*d* hybridization on the formation of SGSs. Calculated atom-resolved DOS of Fe (*a*), Mn (*b*) and Ga (*c*) atoms. The blue curves are for *C*1*_b_*-FeMnGa, the red curves are for the crystal with the Ga atoms removed. The vertical line indicates the Fermi level.

Fig. 7 ▸ shows the atom-resolved DOS of Fe, Mn and Ga atoms for *C*1*_b_* FeMnGa (blue curve) and for FeMn (red curve) with the Ga atom removed from the crystal. Since the Ga atom is removed from the crystal, Fig. 7 ▸(*c*) only gives the DOS of Ga for *C*1*_b_* FeMnGa. In the FeMn crystal hypothetical alloy (red curve), there is a gap in the spin-down channel for DOS of both Fe and Mn, and *E_F_* is located at the gap in the spin-down bands and crosses a peak in the spin-up bands. As Ga atoms are added to the structure, the DOS of Fe and Mn have a totally different distribution which shows a large overlap in energy with the DOS of Ga atoms. This indicates a strong hybridization between the *d* states of the transition metals Fe and Mn, and the *p* states of the main group element Ga. The interaction of FeMn-*d* and Ga-*p* leads to the redistribution of electrons in the FeMn alloy, hence producing the spin-gapless semiconducting property in *C*1*_b_*-ordered FeMnGa alloy.

**Figure 8:**
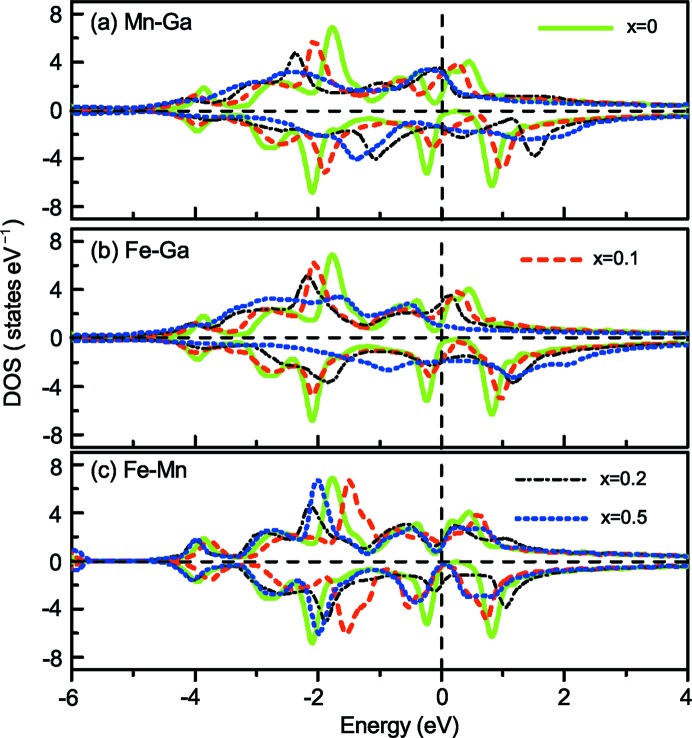
DOS based on the CPA for (*a*) Fe(Mn_1−*x*_Ga_*x*_)(Ga_1−*x*_Mn_*x*_), (*b*) (Fe_1−*x*_Ga_*x*_)Mn(Ga_1−*x*_Fe_*x*_) and (*c*) (Fe_1−*x*_Mn_*x*_) Mn_1−*x*_Fe_*x*_) Ga disorder.

The *C*1*_b_* structure has high atomic order, while atomic disorder is sometimes inevitable in Heusler alloys. Such structural details were treated by applying the CPA. Three atomic disorders, *i.e.* Fe–Ga, Mn–Ga and Fe–Mn were considered in the present work. The results are given in Fig. 8 ▸. It can be seen that the DOS exhibits SGS properties when *x* = 0. With the increase in the degree of disorder of Fe–Ga, Fe–Mn or Mn–Ga, the SGS properties gradually disappear. The SGS properties were totally destroyed when *x* = 0.5, especially for MnGa and FeGa disorder. This indicates that the distribution of DOS is very sensitive to atomic disorder, which reduces hybridization between the *d* states of the transition-metal atoms resulting in smaller exchange splitting of the *d* band (Özdoğan & Galanakis, 2011 ▸). It should be noted that the DOS of ordered FeMnGa (*x* = 0) based on the KKR-CPA has a smaller energy gap with respect to the results based on the DFT-GGA (as shown in Fig. 6 ▸). Similar cases also exist in the well known SGS Mn_2_CoAl (Liu *et al.*, 2008 ▸; Xu *et al.*, 2014 ▸). The important conclusion, however, is that the atomic disorder has a crucial influence on electronic structure. Therefore, highly ordered *C*1*_b_* structure is important for obtaining SGS properties.

FeMnGa alloys can crystallize in different structural phases when treated under different conditions. The B2 phase can be obtained with annealing at 1073–1373 K (Zhu *et al.*, 2009 ▸; Yang *et al.*, 2016 ▸). The highly ordered *C*1*_b_* (or *L*2_1_) phase is usually obtained from the B2 phase by ordering treatment (Ishikawa *et al.*, 2008 ▸). An Ni_3_In type hexagonal phase was recently observed with annealing at 873–923 K (Okumura *et al.*, 2014 ▸; Liu *et al.*, 2018 ▸). The order–disorder transition temperature from the B2 to *C*1*_b_* (or *L*2_1_) phase in Heusler alloys is always between 773 and 973 K (Ishikawa *et al.*, 2008 ▸). Therefore, it is necessary to carefully avoid the hexagonal phase when obtaining the *C*1*_b_* phase in experiment.

### 3.3. Isoelectronic substitution   

**Figure 9:**
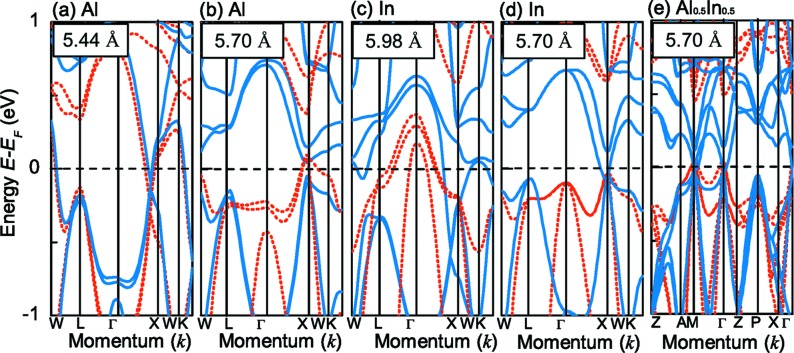
Band structures for FeMnAl with lattice parameters at 5.44 Å (*a*) and 5.70 Å (*b*), for FeMnIn at 5.98 Å (*c*) and 5.70 Å (*d*), and for FeMnAl_0.5_In_0.5_ at 5.70 Å (*e*) based on the GGA.

Since FeMn*Z* (*Z* = Al, In) have the same valence electrons as FeMnGa, they meet the requirement of fully compensated ferrimagnetism according to the Slater–Pauling rule. Therefore, we also performed calculations on these alloys. The optimized equilibrium lattice constants for FeMnAl and FeMnIn are 5.44 and 5.98 Å, respectively. Table 2 ▸ gathers their corresponding atomic and total spin magnetic moments. The moments of the Al and In atoms are negligible. The Fe and Mn atoms contribute the main local magnetic moments and are arranged antiparallel to each other. This leads to a small non-zero net spin magnetic moment deviating from the Slater–Pauling rule, which indicates the loss of SGS properties. The corresponding band structures for FeMnAl and FeMnIn are present in Fig. 9 ▸. The band structures represented in Figs. 9 ▸(*a*) and 9 ▸(*c*) are with equilibrium lattice constants. No SGS properties are observed. Both the magnetic moment and band structures indicate that FeMnAl and FeMnIn in the ground state are not FCF-SGSs. However, by correcting the lattice constants of FeMnAl and FeMnIn, the Fermi energy would again move into the gap. As shown in Figs. 9 ▸(*b*) and 9 ▸(*d*), expansion and contraction of the lattice constants can induce SGS electronic structures in FeMnAl and FeMnIn, respectively, and we can see that both FeMnAl and FeMnIn present FCF-SGS for the specific lattice constant of 5.70 Å. This means that the lattice constant of 5.70 Å is a key point for realising FCF-SGS. The problem is that expansion of 4.78% for FeMnAl and contraction of 4.68% for FeMnIn are too large to realize in practice. A more feasible way to modulate the lattice constant experimentally is to isoelectronically replace the *sp* element with an element in the same main group. This will effectively change the lattice constant because of the different atomic radii, thereby adjusting the energy gap without altering the number of valence electrons. Based on the calculations, we found that the optimized equilibrium lattice constant of FeMnAl_0.5_In_0.5_ is exactly 5.70 Å, and the band structure exhibits the idealized SGS behaviour as shown in Fig. 9 ▸(*e*), the magnetic moment of which is 0 *μ_B_* as shown in Table 2 ▸. Therefore, substituting 0.5In for Al may realize FCF-SGS properties in its ground state in FeMnAl (In).

## 4. Conclusions   

In this work, we employed first-principles calculations to investigate the crystal structure, electronic and magnetic properties of *C*1*_b_*-ordered stoichiometric half Heusler FeMnAl/Ga/In alloys. Our calculations show that the band structure of FeMnGa exhibits a considerable gap in one of the spin channels and a zero gap in the other thus allowing for higher mobility of fully spin-polarized carriers. The band gap originates from the strong hybridization between the *d* states of the transition-metal atoms. The interaction of FeMn-*d* and Ga-*p* leads to the redistribution of the electronic states of the Fe and Mn atoms, which is very important for the formation of SGS properties. The localized magnetic moments of the Fe and Mn atoms have an antiparallel arrangement leading to a fully compensated ferrimagnetism. The exact cancellation of the magnetic moment originates from the antiparallel arrangement of inequivalent magnetic ions (Fe and Mn) being totally different from that of conventional antiferromagnets. One of the most important features is that the electronic structure for the two spin directions is completely asymmetrical with respect to the spin direction, which comes from the asymmetrical distributions of the electronic states of the Fe and Mn atoms. These results indicate that the *C*1*_b_*-ordered stoichiometric FeMnGa is an HM-FCF with a spin-gapless semiconducting nature. The highly ordered *C*1*_b_* structure is very necessary because the SGS properties are very sensitive to atomic disorder. Similar band dispersion and compensated moments were found in *C*1*_b_*-ordered half Heusler FeMnAl_0.5_In_0.5_ alloys. This work is expected to enrich the diversity of spin-polarized antiferromagnetic materials and boost the development of spintronics.

## Figures and Tables

**Table 1:** Inequivalent site occupancies within the *C*1*_b_*-type structure Void means that no atoms occupy the site.

	A (0 0 0)	B (0.25 0.25 0.25)	C (0.5 0.5 0.5)	D (0.75 0.75 0.75)
Type I	Fe	Mn	Void	Ga
Type II	Mn	Fe	Void	Ga
Type III	Fe	Void	Mn	Ga

**Table 2:** Total and atom-resolved spin magnetic moments in *μ_B_* for the FeMn*Z* alloys, where *Z* = Al, Ga, In or In_0.5_Al_0.5_, as a function of the lattice constants PCLC represents the percentage change of lattice constant with respect to those at equilibrium. Negative (or positive) signs of PCLC means the uniform strain is a contraction (or expansion).

Compounds	*a* (Å)	PCLC (%)	Fe	Mn	*Z*	Total
FeMnGa	5.55	0.91	−1.92	2.04	−0.11	0.00
	5.50	0.00	−1.68	1.78	−0.10	0.00
	5.45	−0.91	−1.40	1.53	−0.08	0.05
	5.35	−2.70	−0.78	0.91	−0.06	0.06
FeMnAl	5.44	0.00	−0.74	0.91	−0.06	0.11
	5.70	4.78	−2.24	2.38	−0.14	0.00
FeMnIn	5.98	0.00	−2.93	3.70	−0.15	0.71
	5.70	−4.68	−2.21	2.33	−0.12	0.00
FeMnIn_0.5_Al_0.5_	5.70	0.00	−2.34	2.49	−0.15	0.00
